# Design and validation of an oligonucleotide microarray for the detection of genomic rearrangements associated with common hereditary cancer syndromes

**DOI:** 10.1186/s13046-014-0074-9

**Published:** 2014-09-11

**Authors:** Debora Mancini-DiNardo, Thaddeus Judkins, Nick Woolstenhulme, Collin Burton, Jeremy Schoenberger, Matthew Ryder, Adam Murray, Natalia Gutin, Aaron Theisen, Jayson Holladay, Jonathan Craft, Christopher Arnell, Kelsey Moyes, Benjamin Roa

**Affiliations:** Myriad Genetic Laboratories, Inc., Salt Lake City, UT 84108 USA

**Keywords:** Microarray analysis, Large genomic rearrangements, Hereditary breast and ovarian cancer, Lynch syndrome, Genetic screening

## Abstract

**Background:**

Conventional Sanger sequencing reliably detects the majority of genetic mutations associated with hereditary cancers, such as single-base changes and small insertions or deletions. However, detection of genomic rearrangements, such as large deletions and duplications, requires special technologies. Microarray analysis has been successfully used to detect large rearrangements (LRs) in genetic disorders.

**Methods:**

We designed and validated a high-density oligonucleotide microarray for the detection of gene-level genomic rearrangements associated with hereditary breast and ovarian cancer (HBOC), Lynch, and polyposis syndromes. The microarray consisted of probes corresponding to the exons and flanking introns of *BRCA1* and *BRCA2* (≈1,700) and Lynch syndrome/polyposis genes *MLH1*, *MSH2*, *MSH6*, *APC*, *MUTYH*, and *EPCAM* (≈2,200). We validated the microarray with 990 samples previously tested for LR status in *BRCA1, BRCA2*, *MLH1*, *MSH2*, *MSH6*, *APC*, *MUTYH*, or *EPCAM*. Microarray results were 100% concordant with previous results in the validation studies. Subsequently, clinical microarray analysis was performed on samples from patients with a high likelihood of HBOC mutations (13,124), Lynch syndrome mutations (18,498), and polyposis syndrome mutations (2,739) to determine the proportion of LRs.

**Results:**

Our results demonstrate that LRs constitute a substantial proportion of genetic mutations found in patients referred for hereditary cancer genetic testing.

**Conclusion:**

The use of microarray comparative genomic hybridization (CGH) for the detection of LRs is well-suited as an adjunct technology for both single syndrome (by Sanger sequencing analysis) and extended gene panel testing by next generation sequencing analysis. Genetic testing strategies using microarray analysis will help identify additional patients carrying LRs, who are predisposed to various hereditary cancers.

## Introduction

Sequencing and large rearrangement (LR) analyses detect DNA changes within hereditary cancer genes and are offered to individuals with a personal and/or family history of cancer to identify pathogenic mutation carriers. For example, patients with pathogenic mutations in *BRCA1* or *BRCA2* have a diagnosis of hereditary breast and ovarian cancer syndrome (HBOC), a condition for which there are extensive medical management guidelines aimed at the prevention and early detection of breast and ovarian cancer. Lynch syndrome is the most common inherited colorectal cancer (CRC) syndrome and is associated with mutations in DNA mismatch repair (MMR) genes, mainly *MLH1* and *MSH2*, but also *MSH6*, *PMS2*, and *EPCAM* [1]. In addition, there is an increased risk for colorectal cancer associated with mutations in the *APC* (familial adenomatous polyposis [FAP] or attenuated-FAP) and *MUTYH* (*MUTYH*-associated polyposis) genes. Screening is important for genetic counseling in affected families and for early diagnosis or disease prevention in carriers. Early identification of mutation carriers allows for increased clinical surveillance and early detection, and may prompt more aggressive prevention strategies, such as prophylactic surgery or chemoprevention, to reduce risk.

Conventional Sanger sequencing detects the majority of germline mutations associated with hereditary cancers, which consist primarily of nonsense, missense, small in/del and splice-site mutations. However, conventional Sanger sequencing cannot detect certain classes of genetic alterations, including single- and multi-exonic genomic rearrangements such as deletions and duplications. Such genomic rearrangements account for a clinically significant proportion of the common hereditary cancer syndromes. For instance, genomic rearrangements, primarily deletions, account for 5-30% and 10-60% of all *MLH1* and *MSH2* pathogenic germline alterations, respectively, in Lynch syndrome families; the wide range of frequencies is a result of small sample populations in most studies [2-9]. Baudhuin et al. utilized both Southern blotting and MLPA techniques in a consecutive series of 365 unrelated cases and found that, although the majority of pathogenic mutations identified in *MLH1* and *MSH2* were point mutations and small insertions/deletions, large genomic alterations were present in 17.9% and 45.3% of the *MLH1* and *MSH2* mutation-positive carriers, respectively [10]. Large genomic rearrangements in *BRCA1* and *BRCA2* account for a low but persistent percentage of all *BRCA1* and *BRCA2* alterations [11,12]. Genomic rearrangements of *BRCA1* appear to be more prevalent than *BRCA2* genomic rearrangements [13].

A variety of molecular genetic techniques can be used to detect genomic rearrangements in individuals suspected of a hereditary cancer syndrome, including multiplex quantitative PCR, MLPA, and Southern blotting. The sequencing of the human genome and the development of high-throughput methods of robotically arraying genetic material on a solid substrate have led to the development of genomic microarrays [14,15], which represent the integration of traditional and molecular cytogenetic techniques and enable the clinical diagnosis of chromosomal abnormalities at an unprecedented resolution. Microarrays comprise thousands of discrete segments of DNA, or probes, selected from genomic regions of interest, from intervals throughout the genome. Microarray analysis has been successfully used to detect changes in DNA copy number in genetic disorders associated with congenital anomalies and in solid tumors [16-18]. Several studies have used microarrays for the identification of genomic rearrangements associated with common hereditary cancer syndromes in a research setting [19,20]. Staaf et al. designed a custom oligonucleotide microarray for the characterization of genomic rearrangements of *BRCA1, BRCA2*, *MLH1*, and *MSH2*. The microarray results were concordant with MLPA in 26/30 cases; four discrepancies were the result of inaccurate MLPA results.

In this study, we describe the design and validation of an oligonucleotide-based microarray targeted to genes associated with hereditary cancer. Microarray analysis of patients referred to our laboratory for LR testing in conjunction with sequencing analysis demonstrates that LRs represent a substantial proportion of genetic mutations identified in the clinical laboratory. It should be noted that an increasing number of laboratories have integrated the use of next generation sequencing (NGS) technology for the detection of mutations in panels of genes. Though NGS is a proven technology for the accurate detection of sequencing mutations, detection of LRs by NGS remains challenging [21]. Until we achieve accurate and reliable LR detection by NGS, microarray CGH will remain a viable transitional tool for the detection of LRs for both single syndrome and extended panel testing.

## Materials and methods

### Microarray design

Our laboratory developed a high-density oligonucleotide microarray consisting of approximately 1,700 overlapping probes corresponding to exons and flanking introns of *BRCA1* and *BRCA2*. Approximately 2200 overlapping probes correspond to exons and flanking introns of *MLH1*, *MSH2*, *MSH6*, *APC*, and *MUTYH*, and exons 2, 3, 8, 9, and the 3’ UTR of *EPCAM*. On average, each base of every gene on the microarray has 2.5x or greater probe coverage. The probes (≈45-60 bases) are tiled to ensure detection of rearrangements that are as small as a few hundred bases in length. In addition, probes were selected to overlap flanking regions of repetitive sequence (e.g. guanine cytosine (GC)-rich regions). A stringent selection process was used to create an optimal probe set. The probes designed *in silico* were tested empirically against known negative and positive samples to produce a microarray CGH assay with enhanced sensitivity and specificity to detect variations in dosage.

### Microarray analysis

Genomic DNA was labeled with Cy3 & Cy5 dyes using an Enzymatic Labeling Kit (Agilent Technologies, Santa Clara, CA) according to manufacturer’s instructions. Array hybridization and washing were performed on custom Agilent 8×15k arrays (Agilent Technologies), and arrays were scanned using a High Resolution Microarray Scanner (Agilent Technologies). Analysis of the microarray data was performed in two steps. First, a proprietary software prediction program predicted the likelihood of a particular result for a given sample. Specifically, the prediction algorithm examined a window of contiguous probes, a defined subset of which must have had increased or decreased copy number to generate a positive call. Solitary probes that deviated from the baseline did not trigger a positive call by the algorithm. The software analysis was accompanied by visual review of the data.

Briefly, the average ratio (Cy5:Cy3) of fluorescent intensity obtained from cohybridized test and reference DNA for each probe was calculated and normalized. In addition to the normalizations carried out by Agilent’s Feature Extraction, each probe is also normalized based on GC-content. This normalization is not necessary if all probes designed fall within tight GC-content criteria but this would not allow for tiling coverage across many genes. This normalization works by compensating for dosage biases introduced by dye normalization in probes based on their GC-content and signal intensity difference of the Cy3 and Cy5 labeled probes. The average ratios of the fluorescent intensities for each case were converted to a log_2_ scale and plotted using our proprietary software. This software normalizes each probe based on the historic performance of that probe to minimize biases attributable to the test rather than the patient sample. The theoretical log_2_ conversions for ratios (test/reference) of 1/2, 2/2, and 3/2 are approximately −1, 0, and 0.58 respectively. In practice, the actual values never reach their theoretical limits. For single-copy losses (1/2) and single-copy gains (3/2), we used thresholds of approximately −0.5 and 0.29, respectively; deletions are typically visualized as probe clusters that center within the −0.5 to −1.0 amplitude lines. A cluster of probes centered between the 0.29 to 0.58 amplitude lines represents a duplication.

### Microarray validation study

To validate the microarray we performed microarray analysis on samples that were previously identified to be positive or negative for a LR in *BRCA1, BRCA2, MLH1*, *MSH2*, *MSH6*, *EPCAM, APC,* and *MUTYH* by multiplex quantitative PCR, MLPA, Southern blotting or long-range PCR. An additional synthetic control using genomic DNA digested with restriction enzymes was also used to assess *MUTYH*.

### Clinical testing for LRs by microarray analysis

To examine the proportion of LRs among genetic mutations identified by a large clinical laboratory, we performed microarray analysis on patients referred to Myriad Genetic Laboratories, Inc., for clinical testing of *BRCA1* and *BRCA2* (May 2012 to November 2013) or *MLH1*, *MSH2*, *MSH6*, *EPCAM*, and/or *APC* (September 2012 to November 2013). All patient data regarding clinical history and ancestry were obtained by health care provider report on test requisition forms. For each patient population, data were analyzed for patients who met clinical criteria predicting a relatively high probability of carrying a mutation in *BRCA1* or *BRCA2* (HBOC), *MLH1*, *MSH2*, *MSH6* or *EPCAM* (Lynch syndrome) or *APC* (polyposis). These patients received LR analysis concurrent with sequencing analysis. Patients were excluded if microarray and sequencing analysis were not performed concurrently.

## Results

### Validation study

We initially validated the microarray assay for hereditary colorectal cancer genes with a blinded analysis of 357 previously tested DNA samples derived from patients with a personal or family history of CRC, of which 264 were extracted from blood and 93 from buccal samples. We correctly identified all 88 positives among 357 samples that were previously examined for deletions and duplications in *MLH1*, *MSH2*, *MSH6,* and the 3’ terminal region of *EPCAM* by multiplex quantitative PCR and/or MLPA. We also correctly identified all 7 positive samples among 307 that were previously tested for the presence of LRs in *APC* by Southern blot and MLPA. We correctly identified a homozygous *MUTYH* deletion previously detected by sequencing and confirmed by long-range PCR, as well as a synthetic positive control sample tested in replicates.

We next validated the microarray by blinded analysis of DNA samples previously examined for LR mutations in *BRCA1* and *BRCA2* using our clinically validated multiplex quantitative PCR assay. The microarray validation was carried out in two phases and tested a total of 633 DNA samples. The second phase of validation incorporated minor enhancements for process efficiency that did not affect test sensitivity or specificity. The DNA samples examined for LRs were extracted from 317 peripheral blood and 316 buccal mouthwash specimens. Forty-two of the blood samples and 38 of the buccal samples were positive for a LR. Results for all 633 DNA samples were 100% concordant with the original multiplex quantitative PCR result. In addition, these validation studies correctly identified two instances of an Alu insertion in *BRCA2* specific to the Portuguese population (c.156_157insAlu). The microarray assay for *BRCA1, BRCA2, MLH1*, *MSH2*, *MSH6*, *EPCAM*, *APC*, and *MUTYH* was validated against a collective total of 990 DNA samples that were previously characterized for LRs with 100% concordance.

### Performance of microarray in the clinical setting

Once validated, we performed microarray analysis on samples from patients referred for HBOC (13,124), Lynch syndrome (18,498), and polyposis syndrome (2,739) testing. All samples received LR analysis by microarray concurrent with sequencing analysis. Among all Lynch syndrome deleterious and suspected deleterious mutations detected, 17.2% (189/1,098) were LRs in *MLH1*, *MSH2*, *MSH6*, or *EPCAM* compared with 82.8% (909/1,098) sequencing mutations in *MLH1*, *MSH2*, and *MSH6* (Table 1). Deletions in the 3’ terminal region of *EPCAM* that affect expression of the adjacent *MSH2* gene (but not *EPCAM* point mutations) are associated with Lynch syndrome [22,23]. Of the LRs detected, 27.0% (51/189) were in *MLH1*, 57.7% (109/189) in *MSH2*, 5.3% (10/189) in *MSH6*, and 4.2% (8/189) in the 3’ terminal region of *EPCAM* alone. Deletions in both *MSH2* and the 3’terminal region of *EPCAM* comprised 5.8% (11/189) of the LRs. Collectively, 92.1% (174/189) of the LRs were deletions and 7.9% (15/189) were duplications within *MLH1*, *MSH2*, *MSH6*, and *EPCAM*.

**Table 1:** **Clinical testing summary**

**Syndrome**	**Gene(s) tested**	**Patients tested** ^**a**^	**Total positive sequence and LR mutations**	**Total LR (% of positive mutations)**	**LR by gene (% of LR)**
HBOC	*BRCA1*	13,124	2,186	185 (8.5%)	167 (90.3%)
	*BRCA2*				18 (9.7%)
Lynch syndrome	*MLH1*	18,498	1,098	189 (17.2%)	51 (27.0%)
	*MSH2*				109 (57.7%)
	*MSH6*				10 (5.3%)
	*EPCAM*				8 (4.2%)
	*EPCAM/MSH2*				11 (5.8%)
Polyposis	*APC*	2,739	324	31 (9.6%)	31 (100%)

^a^Sequencing and rearrangement testing done concurrently.

Among all *APC* deleterious and suspected deleterious mutations detected, 9.6% (31/324) were LRs while 90.4% (293/324) were sequencing mutations. Of the LRs detected in *APC*, 93.5% (29/31) were deletions and 6.5% (2/31) were duplications.

Of suspected HBOC samples tested, 1.4% (185/13,124) had a LR mutation in *BRCA1* or *BRCA2*. Of all HBOC deleterious or suspected deleterious mutations in *BRCA1* or *BRCA2*, 8.5% (185/2186) of mutations were LRs and 91.5% (2001/2186) were sequencing mutations. Collectively, 76.8% (142/185) of mutations were deletions and 23.2% (43/185) were duplications/triplications within *BRCA1*and *BRCA2*.

## Discussion

We performed targeted microarray analysis concurrent with Sanger sequencing analysis on a large clinical cohort of samples with a high likelihood of HBOC or hereditary CRC mutations and showed that 17.2% of mutations in Lynch syndrome samples, 9.6% of mutations in polyposis samples, and 8.5% of mutations in HBOC samples were LRs. These results demonstrate that LRs constitute a substantial proportion of genetic mutations associated with a common hereditary cancer syndrome identified by a large clinical laboratory.

**Figure 1:**
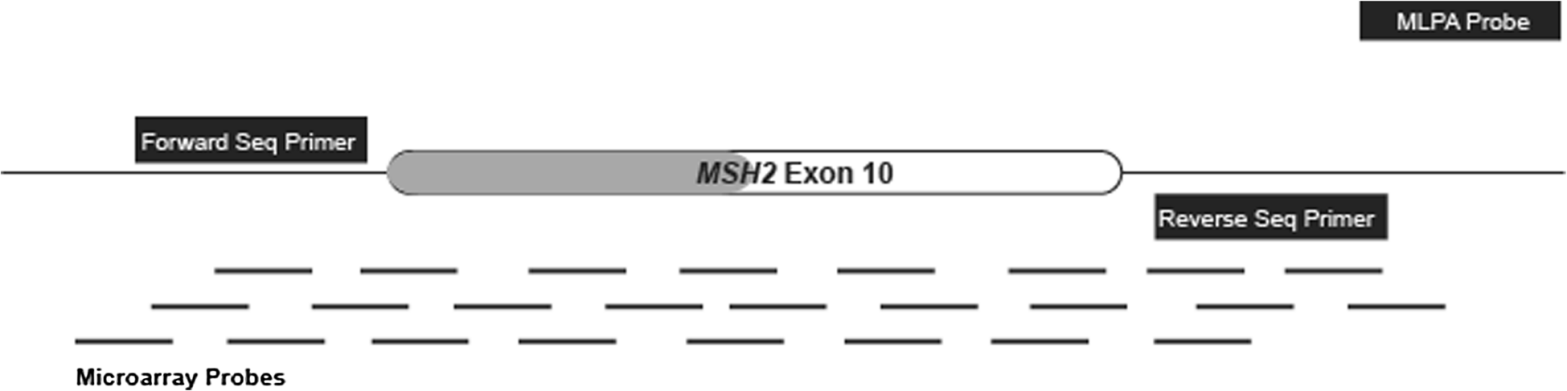
**Diagrammatic representation of the 1900 bp deletion that starts in intron 9 of MSH2 and removes the first 94 bases of exon 10.** This figure maps the locations of multiple overlapping microarray probes that can assess for LRs in this region. The MLH1/MSH2 MLPA kit (MRC-Holland, P003-C1) has only one MLPA probe in intron 10. The locations of the forward and reverse sequencing primers and the microarray probes that cover these regions are also depicted.

The primary advantage of targeted CGH-based microarray analysis is the ability to simultaneously detect exon-level dosage alterations of multiple genes represented on the array. In addition, microarrays have several advantages over other commonly used molecular techniques such as Southern blotting and MLPA. Southern blotting has several limitations: it is time-consuming, expensive and requires a large amount of DNA. MLPA is a relatively inexpensive, simple and reproducible PCR-based method that uses the same equipment used for DNA sequencing [4,24]. MLPA is commonly used for the detection of genomic rearrangements in genes such as *BRCA1* and *BRCA2* [25,26]. However, MLPA has several limitations. Sequence variants in probe binding sites can yield false-positive results; conversely, limited probe coverage across genes may produce false-negative results. Technical artifacts often necessitate confirmatory studies, further increasing cost, resources, and turnaround time. Compared to high-density microarrays, MLPA provides limited information regarding the location of the deletion or duplication breakpoints in the affected flanking/intronic regions, which may lead to laborious mapping for sequence characterization of the rearrangements. Most MLPA reagents are available as kits designed to analyze one or a few genes at a time. The requirement for manual processing further increases sample turnaround time and prohibits high-throughput applications, which are crucial for high-volume sample processing in large clinical laboratories. MLPA also has the potential to miss some partial deletions or duplications. Figure 1 illustrates the extent of probe coverage for *MSH2* exon 10 in our targeted microarray assay. The MLPA assay did not detect this deletion because the single MLPA probe hybridizes to a region of *MSH2* intron 10, which is not deleted. A typical MLPA assay will have 1–2 probes per exon, depending on the size of that exon. Thus, any dosage changes that alter a part of the exon not covered by the MLPA probe will not be detected. Furthermore, it is notable that our targeted microarray probe coverage extends into the binding sites for our sequencing primers, as depicted in Figure 1. In this way, we are able to detect deletions that remove sequencing primer binding sites and lead to mono-allelic sequencing results.

**Figure 2:**
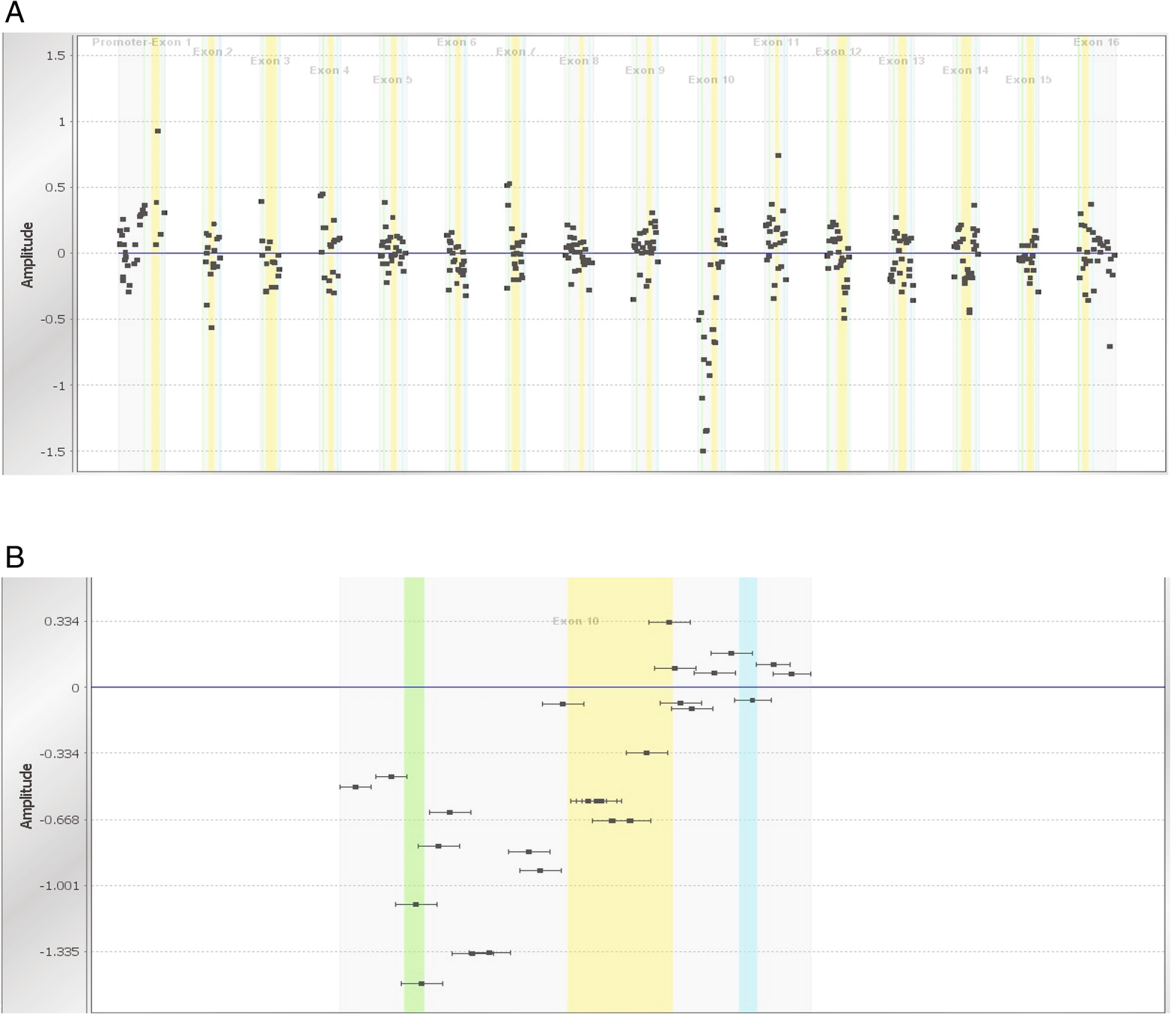
**Targeted microarray result demonstrating a partial deletion of exon 10 in** ***MSH2*** **at the A) gene level and B) probe level.** Vertical yellow lines represent *MSH2* exons. Individual probes are represented by the black dots. Probe clusters for exons devoid of LRs are shown to center at the 0 horizontal line, which represents two allelic copies. The cluster of microarray probes spanning the 5’ portion of *MSH2* exon 10 probe centers at approximately −0.75, indicating the presence of a partial deletion of this exon.

Figure 2 shows the actual partial deletion of exon 10 in *MSH2* in one of our patients, detected by microarray analysis. The microarray showed a loss of probes spanning part of the exon. Confirmatory long-range PCR and sequencing analysis demonstrated that the deletion removed approximately 2,000 base pairs (bp) of sequence (data not shown).

Microarray-CGH is less prone to technical artifacts and is amenable to automation, which allows microarray to be utilized as a high-throughput assay that can decrease sample turnaround times and costs. Microarrays are also scalable—probes can easily be added to current areas of interest and additional syndromes/genes can be added at little additional cost to the laboratory. Targeted microarray CGH therefore provides yet another advantage over other LR detection methodologies. Microarray can be easily adapted for use as an adjunct for panel analysis by NGS, allowing the laboratory to evaluate the LR status of multiple genes at once.

Because microarrays are designed to detect gains and losses in dosage at a specific locus, insertions of material from elsewhere in the genome, particularly of repetitive sequence elements, are difficult to detect. However, our results suggest insertions occur infrequently. To date, the most common of all known insertions found in *BRCA1* and *BRCA2* is an Alu insertion that integrates within nucleotides 156 and 157 in exon 3 of the *BRCA2* gene (c.156_157insAlu). This has been reported as a founder mutation in the Portuguese population. We have specifically designed probes on our microarray assay to screen for the Portuguese founder mutation. We have included probes specific to the native *BRCA2* region in exon 3 that would be disrupted by the insertion as well as other probes that hybridize to the Alu inserted sequence. For this latter probe set, one half of the probe hybridizes to the native *BRCA2* exon 3 sequence and the other half hybridizes to the first 30 bases of the inserted sequence. Together, this collection of probes functions as a screening tool to flag samples that potentially carry the Portuguese insertion. We confirm the presence of the insertion using a targeted breakpoint-specific PCR assay. During the study period, we identified only three cases of the *BRCA2* (c.156_157insAlu) founder mutation in 13,124 clinical samples tested.

Finally, microarrays are not reliant on clinical suspicion of a specific condition. They provide an objective, comprehensive means of genetic diagnosis. In particular, Lynch syndrome can be the most challenging hereditary CRC syndrome to recognize because of the absence of an overt polyposis phenotype, and there are many patients with Lynch syndrome who remain undiagnosed. Testing strategies such as microarray analysis that interrogate LRs will help identify additional patients with a hereditary cancer syndrome, particularly those for whom sequencing analysis was negative.

The ability to analyze multiple genes simultaneously facilitates this shift away from single-locus genetic testing toward panel testing of multiple genes of interest. With the advent of NGS, our laboratory and others are working towards adapting this technology for the detection of LRs as well.

## Conclusion

At present, we have validated the clinical use of microarray CGH to augment LR analysis in our hereditary cancer panel of 25 genes associated with multiple adult-onset hereditary cancers. Once NGS is established as a standard method for the accurate detection of LRs, microarrays may retain utility for confirmatory testing. Panel-based testing strategies will help identify additional patients with a hereditary cancer syndrome and enable clinicians to better manage individuals and families who are affected or at risk for these inherited disorders with specific genetic and clinical counseling, screening, and treatment recommendations.

## Abbreviations

LR: Large rearrangement
HBOC: Hereditary breast and ovarian cancer
CGH: Comparative genomic hybridization
CRC: Colorectal cancer
MMR: Mismatch repair
FAP: Familial adenomatous polyposis
PCR: Polymerase chain reaction
MLPA: Multiplex ligation-dependent probe amplification
NGS: Next-generation sequencing
GC: Guanine cytosine
bp: Base pairs
